# Refining Human Phenotype Ontology (HPO) to enable better phenotype-genotype integration in systemic autoimmune rheumatic diseases

**DOI:** 10.1186/s13023-026-04432-0

**Published:** 2026-06-15

**Authors:** Anastasia-Vasiliki Madenidou, Gillian I. Rice, Sarah Dyball, Ben Parker, Ariane L. Herrick, Hector Chinoy, Adam Stevens, Ian N. Bruce, Tracy A. Briggs

**Affiliations:** 1 Centre for Musculoskeletal Research, The University of Manchester, Manchester, UK; 2 The Kellgren Centre for Rheumatology, Manchester Royal Infirmary, Manchester University NHS Foundation Trust, Manchester, UK; 3 Division of Evolution, Infection and Genomic Sciences, School of Biological Sciences, Faculty of Biology, Medicine and Health, University of Manchester, Manchester, UK; 4 Manchester Centre for Genomic Medicine, Saint Mary’s Hospital, Manchester University Hospitals NHS Foundation Trust, Manchester, UK; 5 Scleroderma and Raynaud’s Research Group, Division of Musculoskeletal and Dermatological Sciences, Faculty of Biology, Medicine and Health, Salford Care Organisation, Northern Care Alliance NHS Foundation Trust, The University of Manchester, Manchester, UK; 6 Centre for Genetics and Genomics Versus Arthritis, Centre for Musculoskeletal Research, Faculty of Biology, Medicine and Health, The University of Manchester, Manchester, UK; 7 National Institute for Health Research Manchester Biomedical Research Centre, University NHS Foundation Trust, The University of Manchester, Manchester, UK; 8 Department of Rheumatology, Salford Royal Hospital, Northern Care Alliance NHS Foundation Trust, Manchester Academic Health Science Centre, Salford, UK; 9 Division of Developmental Biology and Medicine, Faculty of Biology, Medicine and Health, University of Manchester and Manchester Academic Health Science Centre, Manchester, UK

**Keywords:** HPO, Genomics, SARDs, Autoimmunity, WES, Tenosynovitis, Genetics, Phenotype, Scleroderma, Lupus

## Abstract

**Background:**

The Human Phenotype Ontology (HPO) provides a standardised framework for disease-phenotype associations. Given the complexity of systemic autoimmune rheumatic diseases (SARDs) and the absence of prior evaluations, we aimed to assess the completeness of HPO terms capturing SARD-related phenotypic features and SARD-phenotype associations.

**Methods:**

Whole-exome sequencing was performed in 111 individuals from the LEAP (Lupus Extended Autoimmune Phenotype) cohort, a cohort of adult and paediatric SARD patients, who had at least one feature suggestive of a monogenic disease. We evaluated the completeness of HPO annotations relative to documented phenotypic data and SARD diagnoses. We also explored the potential added value of these terms descriptively and by using a phenotype-based genomic tool, Emedgene.

**Results:**

At least one missing HPO term occurred in 36.9% of our patients. Five patient features could not be mapped to existing HPO terms: anti-chromatin antibody, autoimmune hepatitis, scleroderma renal crisis, shrinking lung syndrome, and tenosynovitis. The HPO term “scleroderma” was misdefined, and six HPO terms did not include six linked SARDs in the respective disease-association section. In a random sample of 10 SARD patients, we also demonstrated that HPO terms affect the gene variant prioritisation on the Emedgene platform.

**Conclusions:**

This is the first study to examine the HPO term coverage in SARDs and assess the potential impact of missing terms. We have proposed to the HPO hub the inclusion of absent or misdefined terms and the addition of missing SARD-HPO term associations. We anticipate that expanding the HPO will enhance our ability to characterise the genetic basis of SARDs.

**Supplementary Information:**

The online version contains supplementary material available at 10.1186/s13023-026-04432-0.

## Introduction

The Human Phenotype Ontology (HPO) is a widely adopted, standardised vocabulary for describing medically relevant phenotypes [1], encompassing associations between HPO terms and disease IDs/names from OMIM [2] and Orphanet [3]. This framework enhances genetic diagnosis by enabling computational association between patient phenotypes, disease databases, and gene–phenotype associations [1]. HPO ontology has been initially used for identifying monogenic cases, but more recently has also utilised in genome-wide studies [4, 5]. The ontology has been systematically curated and expanded enhancing its diagnostic utility, but its ability to capture the clinical complexity of systemic autoimmune rheumatic diseases (SARDs) has not been systematically assessed to date [6]. SARDs are rare multi-systemic diseases [7], with a strong genetic basis derived both from rare and common variants [8, 9]. Using the data of a SARD cohort, we aimed to examine the completeness of HPO terms related to SARD phenotypic features and the documented SARD-phenotype associations.

## Methods

The Lupus Extended Autoimmune Phenotype (LEAP) cohort is a UK prospective multicentre study of adult and paediatric patients with a diagnosed SARD (systemic lupus erythematosus (SLE), mixed connective tissue disease (MCTD), systemic sclerosis (SSc), idiopathic inflammatory myopathy (IIM), Sjögren’s disease (SjD) or undifferentiated connective tissue disease (UCTD) (ethical approval: 13/NW/0564). Patients with SLE fulfilled the 2019 European Alliance of Associations for Rheumatology (EULAR)/American College of Rheumatology (ACR) classification criteria for SLE [10], while patients with SSc fulfilled the 2013 ACR/EULAR classification criteria for SSc [11]. Patients with SjD met the 2016 ACR/EULAR classification criteria for SjD [12], and patients with IIM fulfilled the 2017 EULAR/ACR classification criteria for IIM [13]. Patients with MCTD were classified according to the Kasukawa classification criteria for MCTD [14]. UCTD patients did not fulfil the classification criteria for any of the defined CTDs listed above.

Whole Exome Sequencing (WES), using the Illumina NovaSeq6000 [15], was undertaken in 111 patients in the cohort (Table 1) who were considered more likely to have a monogenic cause of disease (namely males, those with paediatric onset disease, suggestive clinical features such as chilblains or cognitive dysfunction, family history, consanguinity or an elevated type I interferon signature [16–18].

**Table 1:** Demographic characteristics of SARD patients undergoing WES for suspected monogenic autoimmunity

	Patients (*n* = 111)
**Age at diagnosis, years**	32.5 (20.5–47.5)
**Age at WES (years)**	49 (30–57)
**Sex (female)**	94 (84.7%)
**Childhood-onset disease**	22 (19.8%)
**Paediatric patients at WES**	10 (9%)
**Ethnicity**	
White	75 (67.6%)
Asian	16 (14.4%)
Black	13 (11.7%)
Other	7 (6.3%)
**Disease**	
SLE	39 (35.1%)
SjD	20 (18%)
MCTD	20 (18%)
UCTD	17 (15.3%)
SSc	10 (9%)
IIM	5 (4.5%)

SARD= Systemic Autoimmune Rheumatic Disease, WES= Whole Exome Sequencing, SLE= Systemic Lupus Erythematosus, UCTD= Undifferentiated Connective Tissue Disease, SjD= Sjogren’s disease SSc=Systemic Sclerosis, MCTD= Mixed Connective Tissue Disease, IIM=Idiopathic Inflammatory Myopathy. Age is median (IQR). Childhood-onset disease was defined as diagnosis before 18 years of age; paediatric patients at WES were those aged under 18 years at the time of WES

FASTQ files and phenotypes mapped to HPO terms [1] were submitted to Emedgene [19]. Emedgene offers phenotype-based variant analysis resulting in list of prioritised variants for each case, labelled as “most likely candidates” and “candidates” [19].

We evaluated the completeness and correctness of HPO annotations in relation to documented phenotypic data and explored whether the missing HPO terms are encompassed by broader existing HPO terms, as well as the potential added value of these terms. We also examined whether known SARD-phenotype associations were included in existing HPO terms. By using Emedgene, we further assessed the potential impact of missing HPO terms on variant prioritisation in a representative sample of 10 SARD patients (approximately 10% of the study population) selected to reflect the full spectrum of the cohort. The sample was chosen to ensure representation across all six SARDs, including both paediatric- and adult-onset cases. We also included individuals with a range of HPO term counts to reflect variability in phenotypic annotation across the cohort.

## Results

The features from 111 patients were mapped to a median number of 13 HPO terms (IQR 8.5–18.0). In total, 132 distinct HPO terms were identified; 23 were recorded only once, while others were mapped in up to 92 patients. All distinct mapped features and their prevalence are presented in the Supplementary Table 1. Of the 111 patients, 41 (36.9%) had one or two missing HPO terms across five of the six SARDs; IIM had none. Five documented features could not be mapped to existing HPO terms: anti-chromatin antibody, autoimmune hepatitis, scleroderma renal crisis, shrinking lung syndrome, and tenosynovitis (Table 2).

**Table 2:** Phenotypic features of 41 out of 111 SARD patients not mapped to HPO terms

Phenotypic features not mapped to HPO terms	Number of patients (total 111)
Anti-chromatin antibody	37 (33.3%)
Autoimmune hepatitis	4 (3.6%)
Scleroderma renal crisis	1 (0.9%)
Shrinking lung syndrome	2 (1.8%)
Tenosynovitis	12 (11%)

SARD= Systemic Autoimmune Rheumatic Disease, HPO= Human Phenotype Ontology

Scleroderma (HP:0100324) is included in the HPO project but is misdefined. Although the autoimmune aetiology of scleroderma is described in the definition, “Pseudoscleroderma”, an umbrella term for conditions characterised by skin fibrosis and mimicking the scleroderma manifestation of SSc and other SARDs, is listed as a synonym [20]. Familial progressive scleroderma (OMIM:181750) and several non-autoimmune diseases, such as porphyria and Werner’s syndrome, appear in the disease-association section, but SSc does not.

We next evaluated the five missing HPO terms, assessing whether they are captured by broader existing terms and determining the potential added value of incorporating more specific terminology. There are distinct HPO terms for the 14 auto-antibodies measured in our cohort, except for anti-chromatin antibody. We used the broader HPO term “autoimmune antibody positivity” (HP:0030057) in our analysis. Anti-chromatin antibody is more prevalent in SLE compared with other SARDs [21], it can be present in SLE in the absence of anti-double-stranded DNA antibody (HP:0020151) [22] and has been linked with disease activity [21].

Although there are nine HPO terms describing various forms of hepatitis, particularly infective aetiologies, autoimmune hepatitis is not included. Autoimmune hepatitis can occur as an isolated condition or in association with SARDs. It was substituted in our analysis by the broader HPO term “hepatitis” (HP:0012115).

Scleroderma renal crisis (SRC) is characterised by the abrupt onset of hypertension (HP:0000822), acute kidney injury (HP:0001919) and microangiopathic haemolytic anaemia (HP:0001937) in the context of SSc [23]. Secondary organ involvement has also been reported in overlap SSc/SLE cases as well as in MCTD [23, 24]. Although there are HPO terms for the components of SRC, there is no HPO term for this characteristic manifestation.

Shrinking lung syndrome is a rare clinical manifestation of SLE, although it has been described in other SARDs [25], characterised by dyspnoea, pleuritic chest pain, and reduced lung volumes [26]. The underlying pathophysiology remains unclear [26], making it challenging to accurately characterise this rare entity using more generic HPO terms, such as dyspnoea (HP:0002094) or pleuritic chest pain (HP:0033771).

“Digital flexor tenosynovitis” (HP:0012276) and not the broader term “tenosynovitis” is an existing HPO term, which would better characterise the involvement of the extensor and flexor tendons in SARDs [27]. The synonym “trigger finger” is included, and the definition is more descriptive of stenosing tenosynovitis/trigger finger rather than of the inflammatory nature of tendon involvement encountered in SARDs and other rheumatic diseases. “Abnormal tendon morphology” (HP:0100261) was used in our analysis, although it is not descriptive of the inflammatory aetiology [28].

To assess the potential impact of missing HPO terms on variant interpretation, we selected a representative sample of 10 patients (approximately 10% of the study population) spanning the full spectrum of SARDs. We included individuals with a range of HPO term counts [7–30] and compared the prioritised variants generated by Emedgene when using and omitting all HPO terms. The number of prioritised variants changed: decreasing in 7 patients (from a mean of 520.3 to 468.4 variants/patient), increasing in 2 patients (from a mean of 518.5 to 634 variants/patient), and was unchanged in 1 patient (215 variants).

Further omissions were identified, with six HPO terms lacking specific SARD entries in their disease-association sections: SLE or SJD from “Increased circulating immunoglobulin concentration (HP:0010702)”, “antiphospholipid syndrome (ORPHA:80)” from “pulmonary embolism (HP:0002204)” and “miscarriage (HP:0005268)”, MCTD, SSc, and IIM from “Nonspecific interstitial pneumonia (HP:0033584)”. SjD from “Enlargement of parotid gland (HP:0011801), and SLE and IIM from “periungual erythema (HP:0033425).

## Discussion

This is the first study to evaluate the HPO term coverage in SARDs. While overall representation of SARD-related features is strong, gaps remain with nearly 40% of our patients having missing terms, including five absent and one misdefined term. Our WES phenotype-based analysis demonstrates that the use of HPO terms clearly impacts the prioritisation of variants as the number of variants changes when HPO terms are added. We have additionally identified a lack of documented association between SARDs and six HPO terms.

As the variant prioritisation analysis was intended as a proof-of-concept to demonstrate whether HPO annotation could influence variant ranking, we considered a representative subset of 10 patients (approximately 10% of the study population) sufficient for this purpose. This subset included patients across all SARDs, both adult and paediatric cases. As patients were mapped to a median of 13 HPO terms (IQR 8.5–18.0), we included individuals with a range of HPO term counts [7–30] to reflect the variability of phenotypic annotation across the cohort. Since changes in variant ranking were observed, supporting the impact of phenotype-driven prioritisation, we did not extend the analysis to the full cohort.

Based on our analysis, we propose the inclusion of all five missing HPO terms, including the term “tenosynovitis” as defined in the OMERACT paper [28], which will enable better phenotypic characterisation of tendon involvement in genomic studies of rheumatic diseases. We also recommend the omission of pseudoscleroderma as a scleroderma synonym and the addition of limited cutaneous systemic sclerosis (ORPHA:220402), diffuse cutaneous systemic sclerosis (ORPHA:220393) and systemic sclerosis (ORPHA:90291) in the disease associations section. A separate HPO term of pseudoscleroderma focusing on sclerosis of the skin outside autoimmunity is suggested. The separation of autoimmune and non-autoimmune related skin sclerosis is more reflective of the pathophysiology of the respective phenotype and will enable a better association of phenotype and related genes in SARDs, such as TREX1 in SSc [29]. The lack of mapped associations between SARDs and existing HPO terms should likewise be rectified. We have already submitted these recommendations to the HPO Hub for consideration (submission to https://github.com/obophenotype/human-phenotype-ontology/issues on 17th November 2025).

The importance of comprehensive HPO term representation for achieving accurate diagnosis through genetic testing has been demonstrated in other conditions. Maassen, et al. collected HPO terms from 98 genetically confirmed Systemic Autoinflammatory Disease patients across eight European centres [6]. Using a computational algorithm, the authors showed that HPO curation improved diagnostic accuracy, increasing the percentage of correct diagnoses from 66% to 86%. In a pilot study, HPO-based WES analysis improved from 10/12 to 12/12 correct diagnoses after curation.

There have been other initiatives to expand and revise the HPO term coverage. In 2019, the ERN-EYE (European Reference Network on rare eye diseases) Ontology Study Group agreed on including 1106 terms relating to ocular phenotypes (HPO) [30]. In 2024, a collaborative initiative expanded the representation of prenatal phenotypes in the HPO [31].

A limitation of this study is that we did not conduct a comprehensive review of all HPO terms and branches relevant to SARDs. Instead, while mapping the clinical features of our WES cohort to HPO terms, we identified missing or misdefined terms and gaps in known disease-phenotype associations, prompting us to focus our investigation on these specific cases. As a result, additional SARD-related features may exist that are either underrepresented or inaccurately represented in the HPO.

## Conclusions

Our findings support the need to expand and revise the HPO by incorporating five underrepresented autoimmune features, correcting one misdefined term and adding known associations between SARDs and HPO terms. As in projects outside rheumatology, their implementation could increase diagnostic yield and further clarify the genetic architecture of SARDs.

## Supplementary Information

Below is the link to the electronic supplementary material.

Supplementary Material 1

## Data Availability

Data are not currently available as LEAP is an ongoing study. Data will be made available upon completion of the study, in accordance with institutional and ethical guidelines.
