# Supplementary material for: Refining Human Phenotype Ontology (HPO) to enable better phenotype-genotype integration in systemic autoimmune rheumatic diseases
Source: Orphanet J Rare Dis. 2026 Jun 15;21:293. doi: 10.1186/s13023-026-04432-0 (PMC13495302; doi:10.1186/s13023-026-04432-0)
Supplement: Supplementary file 1 [file 13023_2026_4432_MOESM1_ESM.docx]

**Supplementary Table 1.** Features of the CTD cohort (n = 111 patients), including clinical manifestations and serological features, were mapped to 132 distinct HPO terms. The table presents each HPO term and its corresponding identifier, together with the number of patients in whom it was observed (prevalence).

| **HPO term** | **HPO identifier** | **Prevalence** |
| --- | --- | --- |
| Abnormal blistering of the skin | HP:0008066 | 1 |
| Abnormal circulating creatine kinase concentration | HP:0040081 | 18 |
| Abnormal circulating globulin concentration | HP:0032179 | 32 |
| Abnormal erythrocyte sedimentation rate | HP:0025021 | 39 |
| Abnormal immunoglobulin level in body fluid | HP:0041076 | 39 |
| Abnormal leukocyte count | HP:0011893 | 19 |
| Abnormal lymphocyte count | HP:0040088 | 34 |
| Abnormal muscle fibre morphology | HP:0004303 | 7 |
| Abnormal pulmonary interstitial morphology | HP:0006530 | 14 |
| Abnormal thrombosis | HP:0001977 | 9 |
| Alopecia | HP:0001596 | 17 |
| Anti-beta 2 glycoprotein I antibody | HP:0032376 | 4 |
| Anti-centromere antibody positivity | HP:0030873 | 5 |
| Anti-complement component C1q antibody positivity | HP:0034077 | 4 |
| Anti-cyclic citrullinated peptide antibody positivity | HP:0034092 | 3 |
| Anti-dsDNA antibody positivity | HP:0020151 | 35 |
| Anti-histidyl tRNA synthetase antibody positivity | HP:0034152 | 2 |
| Anti-MDA5 antibody positivity | HP:0033033 | 1 |
| Anti-Sm antibody positivity | HP:0033040 | 21 |
| Anti-topoisomerase I antibody positivity | HP:0030859 | 5 |
| Anti-U1 ribonucleoprotein antibody positivity | HP:0033028 | 31 |
| Anticardiolipin IgM antibody positivity | HP:0020137 | 2 |
| Antinuclear antibody positivity | HP:0003493 | 62 |
| Antiphospholipid antibody positivity | HP:0003613 | 24 |
| Arterial thrombosis | HP:0004420 | 2 |
| Arthralgia | HP:0002829 | 10 |
| Arthritis | HP:0001369 | 84 |
| Ascites | HP:0001541 | 2 |
| Autoimmune antibody positivity | HP:0030057 | 92 |
| Biliary cirrhosis | HP:0002613 | 1 |
| Calcinosis | HP:0003761 | 3 |
| Chilblains | HP:0009710 | 22 |
| Cognitive impairment | HP:0100543 | 5 |
| Colitis | HP:0002583 | 1 |
| Confusion | HP:0001289 | 4 |
| Cutaneous photosensitivity | HP:0000992 | 35 |
| Decreased circulating complement C3 concentration | HP:0005421 | 20 |
| Decreased circulating complement C4 concentration | HP:0045042 | 34 |
| Diffuse lupus nephritis | HP:0033727 | 2 |
| Digital ischemia | HP:0033402 | 1 |
| Digital pitting scar | HP:0031293 | 8 |
| Digital ulcer | HP:0031917 | 9 |
| Discoid lupus rash | HP:0007417 | 8 |
| Dysphagia | HP:0002015 | 7 |
| Dysphonia | HP:0001618 | 7 |
| Elevated circulating C-reactive protein concentration | HP:0011227 | 22 |
| EMG abnormality | HP:0003457 | 5 |
| Enlargement of parotid gland | HP:0011801 | 6 |
| Episcleritis | HP:0100534 | 1 |
| Fatigue | HP:0012378 | 74 |
| Fever | HP:0001945 | 13 |
| Finger swelling | HP:0025131 | 21 |
| Focal Lupus nephritis | HP:0033731 | 5 |
| Gastroesophageal reflux | HP:0002020 | 15 |
| Glomerular sclerosis | HP:0000096 | 1 |
| Glomerulonephritis | HP:0000099 | 14 |
| Gottron sign | HP:0430033 | 2 |
| Gottron's papules | HP:0025508 | 3 |
| Granular casts | HP:0031201 | 2 |
| Headache | HP:0002315 | 1 |
| Heliotrope rash | HP:0040324 | 3 |
| Haematuria | HP:0000790 | 1 |
| Haemolytic anaemia | HP:0001878 | 6 |
| Hepatitis | HP:0012115 | 2 |
| Hypertension | HP:0000822 | 5 |
| Inflammatory Myopathy | HP:0009071 | 7 |
| Joint swelling | HP:0001386 | 48 |
| Keratoconjunctivitis sicca | HP:0001097 | 32 |
| Keratoconjunctivitis sicca | HP:0001097 | 12 |
| Leukopenia | HP:0001882 | 14 |
| Leukopenia | HP:0001882 | 5 |
| Livedo reticularis | HP:0033505 | 8 |
| Lupus anticoagulant | HP:0025343 | 20 |
| Lupus nephritis | HP:0033726 | 1 |
| Lymphadenopathy | HP:0002716 | 18 |
| Lymphopenia | HP:0001888 | 34 |
| Malabsorption | HP:0002024 | 1 |
| Malar rash | HP:0025300 | 27 |
| Membranous lupus nephritis | HP:0033730 | 2 |
| Memory impairment | HP:0002354 | 1 |
| Mesangial proliferative lupus nephritis | HP:0033728 | 1 |
| Minimal mesangial lupus nephritis | HP:0033729 | 1 |
| Miscarriage | HP:0005268 | 6 |
| Myalgia | HP:0003326 | 3 |
| Myelopathy | HP:0002196 | 1 |
| Myocarditis | HP:0012819 | 1 |
| Myositis | HP:0100614 | 3 |
| Myositis-specific autoantibody positivity | HP:6000678 | 4 |
| Nasal ulcer | HP:0033361 | 5 |
| Nephrocalcinosis | HP:0000121 | 1 |
| Neutropenia | HP:0001875 | 4 |
| Nonspecific interstitial pneumonia | HP:0033584 | 11 |
| Oligoarthritis | HP:0040313 | 5 |
| Oral ulcer | HP:0000155 | 24 |
| Orbital inflammation | HP:6000614 | 2 |
| Pancytopenia | HP:0001876 | 1 |
| Panniculitis | HP:0012490 | 6 |
| Pericarditis | HP:0001701 | 8 |
| Peripheral neuropathy | HP:0009830 | 1 |
| Periungual erythema | HP:0033425 | 2 |
| Pleural effusion | HP:0002202 | 7 |
| Pleuritis | HP:0002102 | 8 |
| Polyarticular arthritis | HP:0005764 | 26 |
| Premature birth | HP:0001622 | 2 |
| Proteinuria | HP:0000093 | 22 |
| Proximal muscle weakness | HP:0003701 | 11 |
| Proximal scleroderma | HP:0550003 | 6 |
| Psychosis | HP:0000709 | 1 |
| Pulmonary arterial hypertension | HP:0002092 | 3 |
| Pulmonary embolism | HP:0002204 | 8 |
| Pulmonary fibrosis | HP:0002206 | 6 |
| Raynaud phenomenon | HP:0030880 | 60 |
| Renal tubular acidosis | HP:0001947 | 1 |
| Rheumatoid Arthritis | HP:0001370 | 3 |
| Rheumatoid factor positive | HP:0002923 | 8 |
| Rheumatoid nodule | HP:6000418 | 1 |
| Scleritis | HP:0100532 | 2 |
| Sclerodactyly | HP:0011838 | 15 |
| Scleroderma | HP:0100324 | 7 |
| Seizure | HP:0001250 | 3 |
| Skin rash | HP:0000988 | 36 |
| Stage 3 chronic kidney disease | HP:0012625 | 1 |
| Systemic lupus erythematosus | HP:0002725 | 39 |
| Telangiectasia | HP:0001009 | 10 |
| Thrombocytopenia | HP:0001873 | 5 |
| Usual interstitial pneumonia | HP:0031950 | 1 |
| Uveitis | HP:0000554 | 1 |
| Vasculitis by anatomical site | HP:0034784 | 4 |
| Vasculitis in the skin | HP:0200029 | 7 |
| Venous thrombosis | HP:0004936 | 10 |
| Weight loss | HP:0001824 | 12 |
| Xerostomia | HP:0000217 | 38 |
